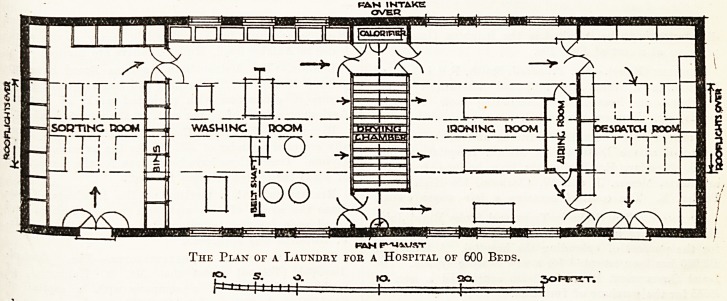# The Planning of Institution Laundries

**Published:** 1912-06-01

**Authors:** 


### HOSPITAL ARCHITECTURE AND CONSTRUCTION.

[Communications on this subject should be marked "Architecture" in the left-hand top corner of the envelope.]

### The Planning of Institution Laundries.

The configuration of the site of an institution will largely affect the position of the laundry block, and also whether it is tc be an isolated block or under the same roof as other departments. The nature of the motive-power for working the various plants will also be a determining factor; but whatever result these several considerations may entail, the fact remains that the ideal laundry should be in principle as like the disinfector as possible. Soiled linen should come in one side, and without any recrossing or overlapping, go out at the opposite side, clean and ready for distribution. The department into which the soiled linen first comes is known as the sorting-room. Here by skilful distribution into separate bins the various classes of napery are sorted out, so that they can be easily removed to the different appliances of the laundry proper.

This is furnished with several types of washing apparatus worked by belt-drives off steam, gas, or electric power. They include washing-machines, wash-troughs, soap-boilers, hydro-extractors, rinsing and blueing troughs, with squeezing-rolls, etc. Whatever apparatus is chosen, it is desirable from every point of view that the various machines should be obtained only from firms of repute.

The clothes having been thoroughly washed and rinsed, if not passed through drying-rollers are taken to the drying-room. This may be described as a chamber where highly tempered air is circulated between and among the clothes, which are hung on clothes-horses; the circulation may be by natural means, but is better if enforced by fans. Probably the most efficient method is that by which air is driven through a steam battery. Where possible it is desirable that the clothes-horses should be drawn out on the side opposite to the laundry. This will be readily followed by a reference to the plan (fig. 1).

The clothes on removal from the drying-horses are received in the ironing-room, where they are dressed either by hand or machine or passed through a mangle. The remark made above regarding the quality of fittings to the washing-room applies with equal force to the ironing-room.

If the clothes are to be passed from the laundry block ready for immediate use they should next pass through an airing-room. This is a chamber heated to an even temperature, to which tempered air is brought and passed quickly through to ensure a thorough airing of the clothes. Passing from here the materials go to the despatch-room, where they are arranged in order and checked previous to being sent to the several blocks from which they originally came.

The whole subject of laundry working is a most consequential one in the well-ordered institution, and it is difficult to lay down hard-and-fast rules for its size, which is relative to the institution and the particular method of arrangement.

Whether certain days are set apart each week for a particular class of work, or whether the different grades of work shall be done simultaneously, will vary according to the instructions and wishes of the superintendent and the nature of the institution. For instance, in the workhouse laundry much of the work will be done by hand labour, which will reduce machinery to a minimum; on the other hand, in the institution, where hand labour means increased cost in staff and maintenance, it will be desirable to instal machinery as far as is compatible with good work.

The plan illustrated shows rooms which might well suit an institution such as a workhouse for, say, 600 beds.

In this article the subject of disinfectors, sterilisers, etc., has not been touched; for though some-

The Plan of a Laundry for a Hospital of 600 Beds.

what allied to the laundry block, it is a separate and distinct problem.

The construction of the hospital building should provide well-lighted airy chambers with impervious walls and floors; probably white glazed brickwork will make the best wall-finish, and concrete for floors and roofs; the floors where water might be splashed should be floated in cement or granolithic mixed with some non-slipping composition, to give a firm foothold. A good quality desiccated pitch pine will make a satisfactory floor for the ironing and despatch rooms. The work which is painted in the laundry, where a more or less steamy atmosphere prevails, should be reduced to a minimum. It is important to specify that the metal pipes, etc., shall be painted in oxides and not in oils.

A feature of such a building as a laundry, where plenty of light and ventilation in the roof are a necessity, is the type of roof-light.

Many patterns of patent roof-lights are on the market; some are of a character requiring annual painting if they are to be kept wholesome, but there are others which have a non-corrosive surface and therefore no maintenance costs. This is the type which actual experience shows, in the long run, to be most economical; the cost is about 11d. per foot super where the span is about five or six feet and the glass ¼-inch rolled cast plate. The introduction of opening lights and crank gears to work them adds somewhat more to the cost as compared with that of other types.

On the plan illustrating this article it is assumed that the power for generating hot water is steam, brought to the calorifier in the form of flow and return from the boilers supplying heat and water to the whole institution.

The æsthetic aspect of the building is ignored in this design. The object is simply to awaken interest in the utilitarian aspect of the problem; but we mention the other, for though the purpose of the building is severely utilitarian, there is no reason why in actuality it should not be pleasing to look at by reason of carefully considered detail and good proportions.

## Figures and Tables

**Figure f1:**